# Preparation of surface plasmon resonance-based nanosensor for curcumin detection

**DOI:** 10.3906/kim-2106-21

**Published:** 2021-09-21

**Authors:** Şebnem ÇIKRIK, Duygu ÇİMEN, Nilay BERELİ, Adil DENİZLİ

**Affiliations:** 1 Department of Bioengineering Division, Institute of Science, Hacettepe University, Ankara, Turkey; 2 Department of Chemistry, Hacettepe University, Ankara, Turkey

**Keywords:** Curcumin, surface plasmon resonance, nanosensor, molecularly imprinting

## Abstract

In this study, the curcumin imprinted and the non-imprinted poly(2-hydroxyethyl methacrylate-N-methacryloyl-L-tryptophan) (poly(HEMA-MATrp)) nanoparticle based surface plasmon resonance (SPR) nanosensors were prepared for the detection of curcumin and characterized by zeta-size analysis, Fourier transform infrared spectroscopy, and scanning electron microscopy. After, the curcumin imprinted and the non-imprinted nanoparticles are attached on the surface of SPR chips. The curcumin imprinted and the non-imprinted SPR nanosensors are characterized by using atomic force microscope, ellipsometer, and contact angle measurements. Kinetic studies were carried out with curcumin aqueous solution at a concentration range of 0.01–150 mg/L using the curcumin imprinted and the non-imprinted SPR nanosensors. In all kinetic analysis, the response time is 14 min for equilibration, adsorption, and desorption cycles. The limit of detection and limit of quantification for the curcumin imprinted SPR nanosensors was 0.0012 mg/L and 0.0040 mg/L, respectively. The validity of the curcumin imprinted SPR nanosensors in real samples was carried out using liquid chromatography-tandem mass spectrometry (LC-MS).

## 1. Introduction

Curcumin is derived from rhizomes of Curcuma longa. Curcumin is used in several areas, which are food area such as spice or colouring substance and herbal medicine area such as therapeutic agents [1]. It is composed of 94% curcumin(Curcumin longa) and the remaining components are known as curcuminoid. Curcumin is the primary curcuminoid in Turmeric and the compound in which most studies are performed [2]. In recent years, researches have been concentrated on curcumin because of its great interest and use in the treatment of various disease such as cancer, Alzheimer’s disease, Crohn’s disease, diabetic, neurological disorders, and autoimmune diseases [3–5]. Although curcumin has so many advantages, it also has limitations of curcumin such as its low bioavailability, rapid elimination, and poor solubility in water. On the other hand, it is well soluble in solvents such as acetone, ethanol, tetrahydrofuran, acetylacetone, chloroform, acetic acid, benzene and toluene, especially methanol. Curcumin is also unstable at basic pH and hydrolytic degradation has been reported even in in-vitro physiological conditions (phosphate buffer, pH = 7.4) [6,7]. Nowadays, curcumin is used in different technological studies. Of these, sensors are used for detection of oligomeric amyloid-beta (Aβ) peptidesat Alzheimer’s disease, at fluometric devices for measurement curcumin at organic solvents, chemosensor for mercury determination in water samples using curcumin nanoparticles [8,9]. High-performance liquid chromatography (HPLC), mass spectrometry, flowcytometry, and electron microscopy are all popular methods for detecting curcumin [10–16]. Although these techniques are very selective, they do not provide fast detection. In the presence of samples which are containing curcumin and analogs, data analysis becomes extremely difficult. Furthermore, using these instruments in a technical laboratory setting requires skilled staff and costly instrumentation [17].

Surface plasmon resonance (SPR) nanosensors, as optical sensor, is a simple and direct technique by measuring the change in refractive index near the metal surface, which is usually gold or silver [18]. By examining the binding speed and binding strength of molecules with SPR nanosensors, a lot of information is obtained on the specificity, molecular interaction, kinetics and affinity of the bond. SPR nanosensor has become very popular nowadays due to its accuracy and sensitivity. By using the SPR system, nanosensors can be developed against many different target molecules and different interactions can be followed. SPR systems are more faster, sensitive and selective applications than other nanosensors. Especially, SPR nanosensors are used in life sciences, pharmaceutical, food, and nanotechnology studies [19–21]. In this study, we take the advantage of using molecular imprinting technique and SPR nanosensor systems advantages for detection of curcumin.

Molecularly imprinting technique (MIT) is the most popular technique for sensitivity and selectivity to determine recognition elements in sensor systems [22–24]. MIT is based on the functional complexes form of functional monomers around a template molecule by covalent or non-covalent interactions, and subsequently to create molecular imprinted polymers (MIPs) with chemical function in a suitable process. Recently MIPs are used to increase biorecognition on molecular surfaces at SPR systems. They have important advantages such as easiness in preparation and being cheaper than other techniques [25,26].

In this study, the curcumin imprinted and the non-imprinted poly(2-hydroxyethyl methacrylate-N-methacryloyl-L-tryptophan) (poly(HEMA-MATrp)) nanoparticles were synthesized and attached on the surface of SPR chip for detection of the curcumin both in aqueous solution and real samples. The curcumin imprinted (MIP) and the non-imprinted (NIP) poly(HEMA-MATrp) nanoparticles were arranged by two-phase mini-emulsion polymerization method. The non-imprinted curcumin nanoparticles were also synthesized using the same method without added curcumin. The non-imprinted nanoparticles were used as a control experiment. After synthesis and characterization of the curcumin imprinted nanoparticles, it was attached to the surface of SPR chip and characterized by atomic force microscope, ellipsometer, and contact angle measurements. The curcumin imprinted SPR nanosensors were performed a real-time detection of curcumin with kinetic analysis at different concentrations, selectivity, and repeatability studies. The repeatability studies of the curcumin imprinted SPR nanosensors were also examined. Also, the extracted curcumin from the tea and spice samples were analyzed by SPR system and liquid chromatography-tandem mass spectrometry (LC-MS).

## 2. Materials and Methods

### 2.1. Materials

Curcumin (CUR), tartrazine (TA), sunset yellow (SY), ethylene glycol dimethacrylate (EGDMA), polyvinyl alcohol (PVA), 2-hydroxyethyl methacrylate (HEMA), ammonium persulfate ((NH_4_)_2_S_2_O_8_), sodium bicarbonate (NaHCO_3_), sodium bisulfite (NaHSO_3_) and sodium dodecyl sulfate (SDS) were obtained from Sigma-Aldrich (St. Louis, Missouri, USA). SPR bare gold chips (SPRchipTM, Masidon, WI, USA) were supplied for the SPRimager II instrument by GWC Technologies (Masidon, WI, USA).

### 2.2. Preparation of The Curcumin Imprinted and Non-Imprinted Nanoparticles

**Figure 1:**
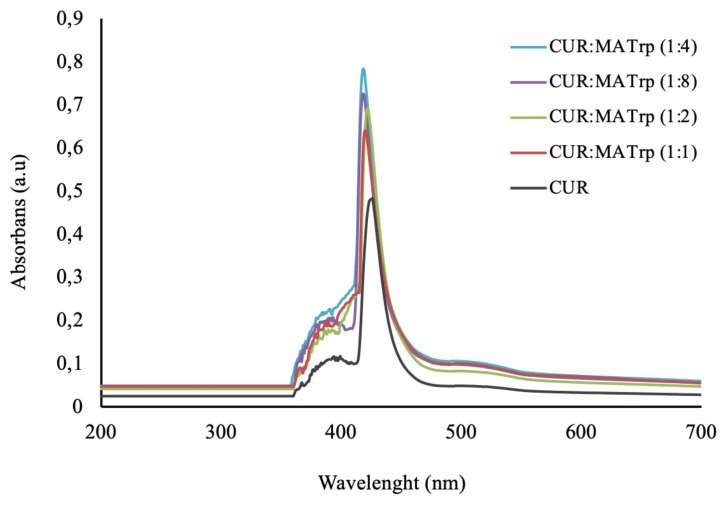
UV-spectrum of CUR:MATrp pre-polymerization complex.

Curcumin, which is used as a template molecule, was dissolved in N-methacryloyl-L-tryptophan methyl ester (MATrp) monomer at different molar ratio for pre-polymerization complex was prepared. N-methacryloyl-L-tryptophan methyl ester (MATrp) which was synthesized and reported previously by Denizli et al. was used in the synthesis of molecular imprinting polymers as functional monomer [27]. For this, the pre-polymerization complex solutions were obtained by adding MATrp at the molar ratios of 1, 2, 4, and 8 M at the 1 M curcumin solution. The absorbance of CUR:MATrp was measured by UV-VIS spectrophotometer (SHIMADZU UV-1601 model, Tokyo, Japan) in the range of 200–700 nm wavelength. The highest absorbance value was obtained at curcumin solution in the molar ratio of 1:4 M for curcumin imprinted nanoparticles synthesis (Figure 1).

After the preparation of the pre-polymerization complex solution, 200 mg of PVA, 30 mg of SDS, and 25 mg of sodium bicarbonate were mixed in 10 mL of water in a magnetic stirrer in the preparation of the first phase. In the next step, 2.1 mL crosslinker EGDMA, 0.45 mL HEMA, and pre-polymerization complex were used in the liquid phase called the oil phase. The oil phase was added to the first phase and homogenized for 30 min at 24.000 rpm. The other aqueous phase, 100 mg PVA and 200 mg SDS were dissolved in 200 mL water and added to the mixture and mixed for 15 min in the sonicator. This mixture was then added to the polymerization device. Finally, 230 mg of sodium bisulfite and 252 mg of ammonium persulfate were added to as polymerization activators and polymerization process was carried out in the reactor with 450 rpm at 40 °C for 24 h. The non-imprinted nanoparticles were synthesized under the same experimental conditions without adding curcumin. The synthesized curcumin imprinted (MIP) and non-imprinted (NIP) nanoparticles were removed from unreacted monomers by washing the deionized water and water/ethanol mixture.

### 2.3. Preparation of SPR Nanosensors

**Figure 2:**
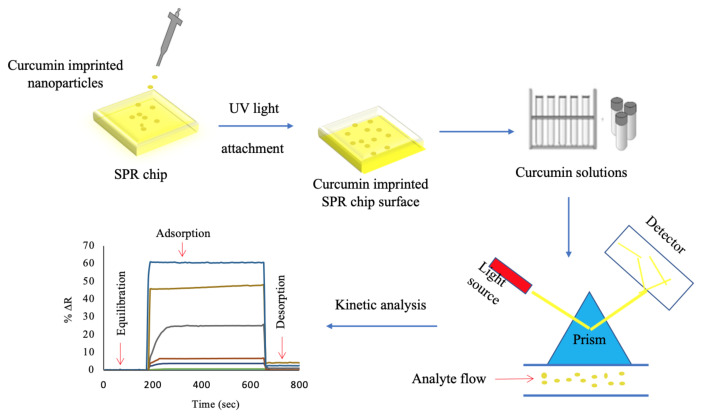
Schematic view of the preparing curcumin imprinted SPR nanosensor.

First of all, allyl mercaptan (C_3_H_6_S) and its thiol (-SH) groups were formed to prepare the surface of the SPR chip and surface modification was performed. The gold chip surface was cleaned with 20 mL acidic piranha solution as mixture ratio of 3:1(v/v) of H_2_SO_4_ and H_2_O_2_ for 5 min and then washed with ethyl alcohol. After washing, it was dried in a vacuum oven at 200 mmHg pressure and 40 °C for 3 h. Finally, 5 μL of allyl mercaptan was dropped on the surface of SPR chips and incubated in the overnight at room temperature. The surfaces of the SPR chips were then washed with ethanol to remove unbound molecules. Then, 4 μL of the curcumin imprinted nanoparticles was dropped on the SPR chip surface using spin coater (LAURELL, WS 650Mz-23NPP, USA). The curcumin imprinted and non-imprinted nanoparticles were attached to the surface of SPR chip under ultraviolet light (365 nm, 100 W) for 20 min and incubated in an oven overnight at 40 °C to stabilize attachment. The preparation of the curcumin imprinted SPR chip was shown in Figure 2.

### 2.4. Characterization Studies

The characterization studies of the curcumin imprinted poly(HEMA-MATrp) nanoparticles were made prior to the preparation of the curcumin imprinted and the non-imprinted SPR nanosensors. The characterization studies of the curcumin imprinted and the non-imprinted poly(HEMA-MATrp) nanoparticles were performed by zeta-sizer (Nano-ZS, Malvern Instrument Company, UK), electron microscope scanning (SEM, Quanta 400F Field Emission, USA) and Fourier transform infrared spectroscopy (FTIR, Thermo Fisher Scientific, Nicolet iS10, Waltham, MA, USA). The size analysis of the curcumin imprinted nanoparticles was performed by Zetasizer device. For this purpose, 1.0 mL of the nanoparticle solution was transferred to the quartz cuvette, the measurement was repeated 3 times. Fourier transform infrared spectroscopy (FTIR) spectrums of the curcumin imprinted (MIP) and non-imprinted (NIP) poly(HEMA-MATrp) nanoparticles were obtained by using a FTIR spectrophotometer (Varian FTS7000, Palo Alto, CA). For this, nanoparticles were dried and then mixed with IF grade KBr. Well mixed sample was pressed into a pellet form and FTIR spectrum of the nanoparticles was recorded.

After characterizing the curcumin imprinted and the non-imprinted nanoparticles, the characterization studies of the curcumin imprinted and the non-imprinted SPR nanosensor surfaces were examined by atomic force microscope, ellipsometer, and contact angle measurements. The surface morphology of the curcumin imprinted and non-imprinted SPR nanosensor surfaces was examined by using an atomic force microscope (Nanomagnetics Instruments, Oxford, UK) in a tapping mod with 5μm×5μm area samples at 1 μm/s scanning speed and 256×256 pixel resolution. For surface thicknesses of the curcumin imprinted and the non-imprinted SPR nanosensor surfaces, EP3-Nulling Ellipsometer (Germany) device was used and the measurements were repeated at 6 different points at 532 nm wavelength. For the hydrophobic characterization of the bare gold SPR chip surface, allyl mercaptan modified SPR chip surface, the non-imprinted and the curcumin imprinted SPR nanosensor surfaces, the contact angle was characterized using the KRUSS device (Hamburg, Germany). The hydrophobicity of the SPR chip surface was determined by using the sessile drop method to drop water on various areas of the SPR chip surface.

### 2.5. Kinetic Analyses

After the characterization studies of the curcumin imprinted (MIP) and the non-imprinted (NIP) SPR nanosensors, kinetic analyzes were performed using SPRimager II to detect curcumin from both aqueous solution and real samples. For kinetic analysis, different curcumin concentrations between 0.01 mg/L and 150 mg/L were prepared in pH 7.4 phosphate buffer. Firstly, phosphate buffer with pH value of 7.4 as equilibration buffer was given to the SPR system for 3 min. Then, curcumin solutions of different concentrations were applied to the SPR system when the system reached equilibrium. The refractive index changes (%DR) were observed in real-time and were performed at 25 °C for 8 min and a flow rate of 2 mL/min. After each kinetic analysis, the desorption step was carried out using 0.5 M NaCI solution for 3 min. In all kinetic analyses, equilibration-adsorption-desorption steps were carried out at 14 min.

In order to evaluate the curcumin imprinted SPR nanosensor selectivity, the responses of SPR nanosensor system against the other competitive molecules tartrazine (TAR) and sunset yellow (SY) were investigated singularly. TAR (MW: 534.36 g/mol) and SY (MW: 452.38 g/mol) dyestuffs have molecular weights and properties that are similar to CUR (MW: 368.38 g/mol). Curcumin, tartrazine, and sunset yellow solutions were prepared with the same concentration (50 mg/L) and their interactions with the curcumin imprinted and the non-imprinted SPR nanosensors were examined separately to compare the selectivity behavior. According to the selectivity studies, the selectivity (k) and relative selectivity (k′) coefficients were calculated in order to compare the results which were obtained with both competitive molecules as sunset yellow and tartrazine. The values k and k′ are described by the following equations [19].


(1) $$\text{k}=\frac{\Delta {\text{R}}_{\text{template}}}{\Delta {\text{R}}_{\text{competitor}}}$$

(2) $${\text{k}}^{\prime }=\frac{{\text{k}}_{\text{MIP}}}{{\text{k}}_{\text{NIP}}}$$

The repeatability studies of the curcumin imprinted SPR nanosensor were carried out using the equilibration, adsorption and desorption cycles five times, with the same curcumin imprinted SPR nanosensor. In order to examine the repeatability of the curcumin imprinted SPR nanosensor, the repeatability studies were made with curcumin at a concentration of 50 mg/L on the same and different days.

### 2.6. Real Sample Analysis

Herbal tea and curcumin powder as real samples were analyzed to determine sensitivity of the curcumin imprinted SPR nanosensors. Firstly, 10 g of herbal tea sample was weighed and was mixed with 50 mL ultrapure water for dissolving. Then, the pH value of the solution was adjusted to 3.0 with acetate buffer. After this step, curcumin was extracted from real samples by micro-extraction method. 75 μL of 1-Butyl-3-methylimidazolium hexafluorophosphate ([BMIM] [PF6]) and 1.5 mL of dichloromethane were added to the mixture as the extraction solution and then mixed in a magnetic stirrer. This extraction solution was placed in an ultrasonic bath heated to 50 °C for 4 min to dissolve the ([BMIM] [PF6]) in the mixture and then kept in an ice-water bath for 10 min. After the centrifugation of tubes at room temperature for 8 min at 4000 rpm, the supernatant portion was removed. Afterwards, the resulting pellet was diluted to 3 mL with ethanol. Spice samples were also prepared using the same method [28].

## 3. Results and Discussion

### 3.1. Characterization Studies

**Figure 3:**
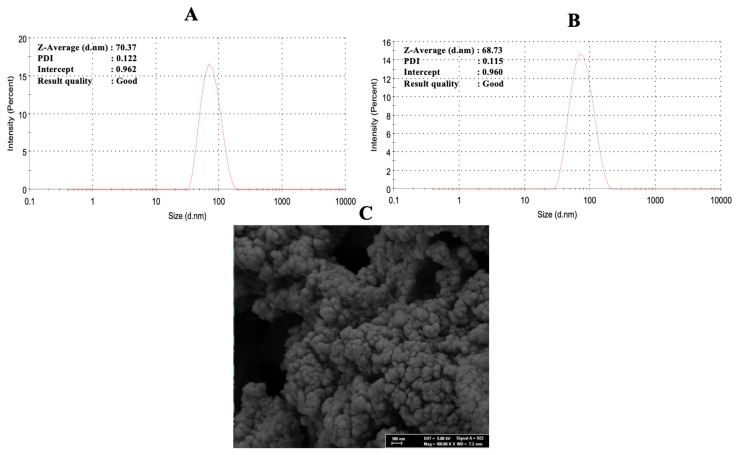
Zeta size measurements (A: curcumin imprinted nanoparticles and B: non-imprinted nanoparticles) and SEM image (C: curcumin imprinted nanoparticles).

The characterization studies of the curcumin imprinted and the non-imprinted nanoparticles were made with zeta-sizer. The average nanoparticle size and polydispersity index (PDI) were measured for the curcumin imprinted nanoparticles using the zeta-sizer. The average size of the curcumin imprinted nanoparticles was measured as 70.37 nm with 0.122 PDI (Figure 3A). Also, the average size of the non-imprinting nanoparticles was measured as 68.73 nm with 0.115 PDI (Figure 3B). SEM analysis of the curcumin imprinted nanoparticles was observed to have a rough surface with a size of about 76.63 nm (Figure 3C).

**Figure 4:**
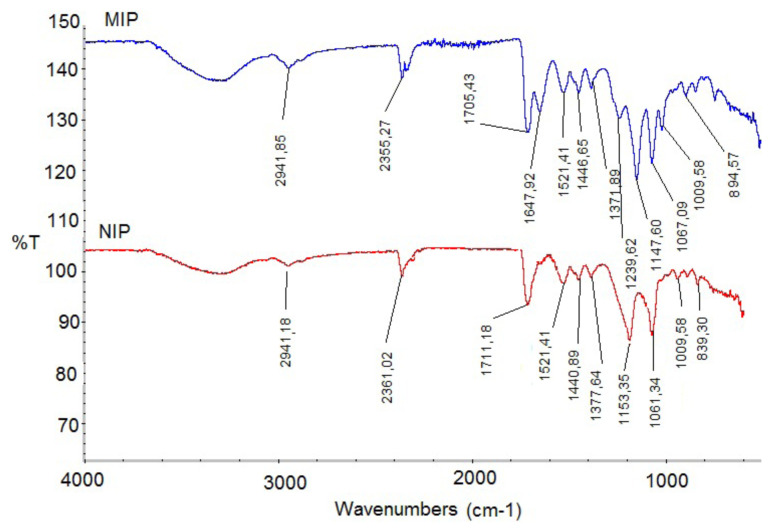
FTIR spectra of the curcumin imprinted (MIP) and non-imprinted (NIP) nanoparticles.

FTIR spectrums of the curcumin imprinted (MIP) and non-imprinted (NIP) poly(HEMA-MATrp) nanoparticles are shown in Figure, respectively. For curcumin imprinted nanoparticles, the band at 1630 cm^−1^ and 1513 cm^−1^ have the C=C and C=O vibration modes. Also, the band at 1239 cm^−1^ was occurred due to the C-H vibrations of aromatic rings. From both spectrum, 2941 cm^−1^ (CH stretching for aliphatic alkyl), 1710 cm^−1^ (C=O stretching), 1446 cm^−1^ and 1371 cm^−1^ (C-N stretching for amide) and 1147 cm^−1^ (aromatic ring bending) bands are quite noteworthy. The existence of some of the functional groups corresponding to these bands within MATrp structure denotes that MATrp monomer was successfully incorporated into nanoparticles structure (Figure 4). The results of the zeta sizer, FTIR, and SEM showed that the molecularly imprinted technique is successful in nanoparticle synthesis.

**Figure 5:**
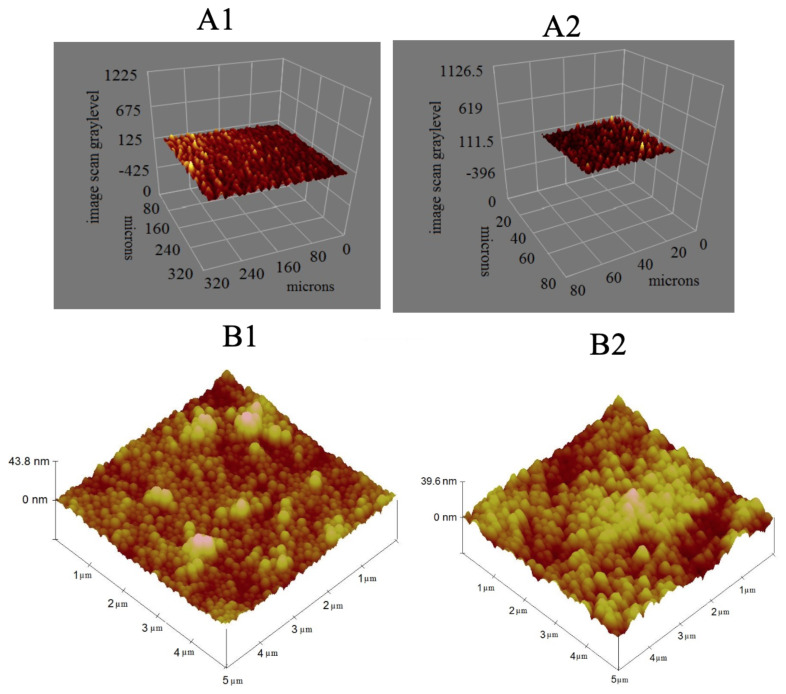
Ellipsometer and AFM images (A1: curcumin imprinted SPR nanosensor surface, A2: non-imprinted SPR nanosensor surface and B1: curcumin imprinted SPR nanosensor surface, B2: non-imprinted SPR nanosensor surface).

The surface characterizations of the curcumin-imprinted and the non-imprinted SPR nanosensors were studied with atomic force microscope (AFM), ellipsometer, and contact angle. According to ellipsometer results, the average thicknesses of the curcumin-imprinted and the non-imprinted SPR nanosensor surfaces were determined as 78.5 nm and 73.4 nm, respectively (Figure 5A1 and 5A2). As can be seen from the ellipsometer results, it was observed that the curcumin imprinted nanoparticles are spread homogeneously on the surface of SPR chip.

Atomic force microscope (AFM) was used for the surface morphology of the curcumin imprinted and non-imprinted SPR nanosensors in tapping mode [29]. The surface depth values of the curcumin imprinted and non-imprinted SPR nanosensor surfaces were 43.8 nm and 39.6 nm, respectively (Figure 5B1 and 5B2). As can be seen from the AFM results, it was observed that the curcumin imprinted nanoparticles were spread homogeneously on the surface of the SPR nanosensor.

**Figure 6:**
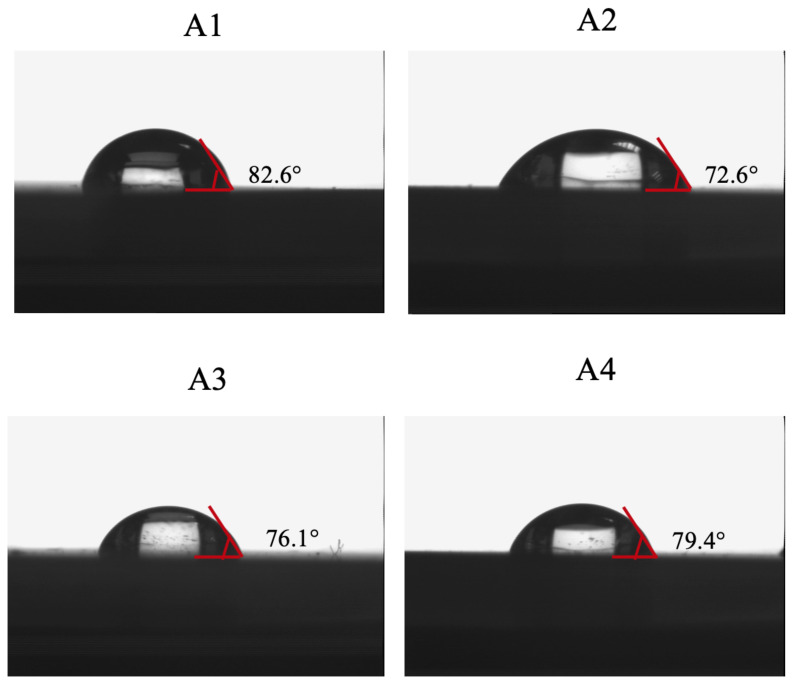
Contact angles (A1: bare gold SPR chip surface, A2: allyl mercaptan modified SPR chip surface, A3: non-imprinted SPR nanosensor surface and A4: curcumin imprinted SPR nanosensor surface).

The hydrophobic property of the bare gold SPR chip surface, allyl mercaptan modified SPR chip surface, the curcumin imprinted and the non-imprinted SPR nanosensor surfaces were characterized by the contact angle. These measurements were taken with water drops dropped on different points of SPR chip surfaces, which were characterized by measuring the hydrophobic property. After allyl mercaptan modification of the bare gold SPR chip, it was observed that the contact angle of the SPR chip surface decreased from 82.6°±3.6 and 72.6°±2.7, respectively (Figure 6A1 and 6A2). This decrease in the contact angle value indicated that the hydrophilicity of the chip surface increased. The contact angle values for the non-imprinted and the curcumin imprinted SPR nanosensor surfaces were found to be 76.1°±3.5 and 79.4º±2.1, respectively (Figure 6A3 and 6A4). The observed increase in surface contact angles depended on the increase in hydrophobicity of the surface. The increase in the hydrophobic character of the surface is expected due to the hydrophobic functional groups of MATrp monomer.

### 3.2 Kinetic Analyzes

**Figure 7:**
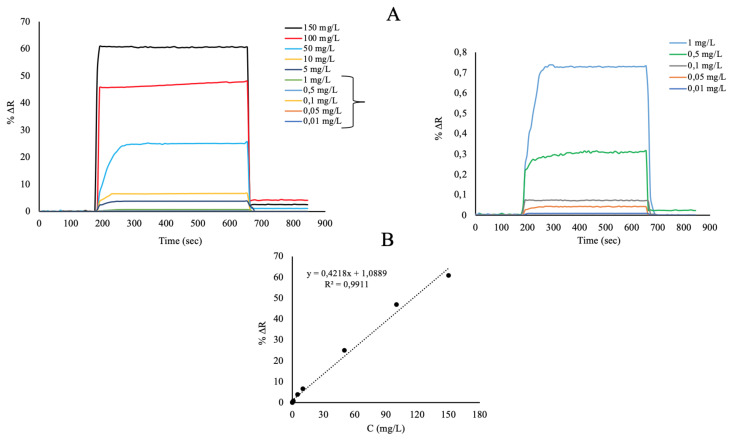
Real-time responses (A) and linear regions (B) of the curcumin imprinted SPR nanosensors in aqueous solutions of curcumin at different concentrations.

In this study, kinetic analyzes were performed by performed using SPRimager II (GWC Technologies, Madison, WI, USA). Curcumin solutions were prepared for use in kinetic studies in phosphate buffer solutions having pH value of 7.4 at concentrations of 0.01–150 mg/L and are given real time values of the SPR sensorgrams in Figure 7A. Firstly, the curcumin imprinted SPR nanosensor was washed with phosphate buffer solution pH 7.4 for 3 min. After, curcumin solutions in the concentration ranges 0.01–150 mg/L were given to the SPRimager II system for 8 min and the resonance frequency change (%DR) was determined for each kinetic analysis. Desorption study was performed with 0.5 M NaCl for 3 min. All kinetic analysis time with the curcumin imprinted SPR nanosensors were completed in approximately 14 min. ΔR (%) based on the signals, kinetic analyzes were obtained and these data were examined with the support of SPRview software program. As shown in Figure 7B, %ΔR value and curcumin concentration increased proportionally. The good linear equation between 0.01 and 150 mg/L concentration is y=0.4218×+1.0889 with a determination coefficient of 0.991. The limit of detection (LOD=3.3S/m) and the limit of quantification (LOQ=10S/m) of curcumin molecules was calculated based on the slope of the calibration curve. The limit of detection and the limit of quantification were calculated with y=0.4218x+1.0889 equation and were determined as 0.0012 and 0.0040 mg/L, respectively. Other sensor studies performed for curcumin determination are given in Table 1 for comparison.

**Figure 8:**
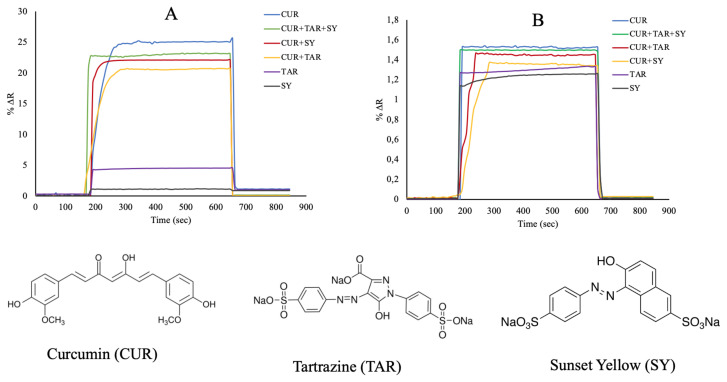
The selectivity studies (A: curcumin imprinted (MIP) and (B) non-imprinted (NIP) SPR nanosensors).

Tartrazine (TAR), sunset yellow (SY) and curcumin (CUR) are the most commonly used colourants in food, cosmetics and medicine. In this study, selectivity analysis of the curcumin imprinted SPR nanosensor was performed using tartrazine and sunset yellow molecules. The mixtures of both curcumin and competitor molecules were prepared, and measurements were taken. The concentration of CUR, TAR and SY were kept constant at 50 mg/L. As seen from Figure 8, there is no specific binding of tartrazine and sunset yellow molecules with the curcumin imprinted nanosensor. Figure 8 showed the responses of the curcumin imprinted SPR nanosensor for TAR and SY were lower than CUR. This is due to the selective cavities of curcumin formed in the curcumin imprinted nanoparticles. The relative selectivity coefficients (k′) results showed that the curcumin imprinted (MIP) SPR nanosensor had higher selectivity for curcumin in comparison to TAR and SY. In addition, the non-imprinted SPR nanosensor was prepared with the curcumin non-imprinted poly(HEMA-MATrp) nanoparticles for using at selectivity studies tartrazine, and sunset yellow solutions were analyzed in the SPR system just like the curcumin imprinted SPR nanosensor. Figure 8 shows that %ΔR value is lower at competitor molecules other than curcumin. The relative selectivity coefficients (k′) of the curcumin imprinted SPR nanosensor for CUR/TAR and CUR/SY were 4.88 and 19.01 times, respectively (Table 2). %DR change of the non-imprinted SPR nanosensor with both curcumin and other competitive agents as TAR and SY were obtained as 1.32 and 1.25, respectively. When the selectivity results are examined, it shows that the curcumin imprinted SPR nanosensors have higher selectivity than the non-imprinted SPR nanosensors.

### 3.3. Real Sample Analysis

**Figure 9:**
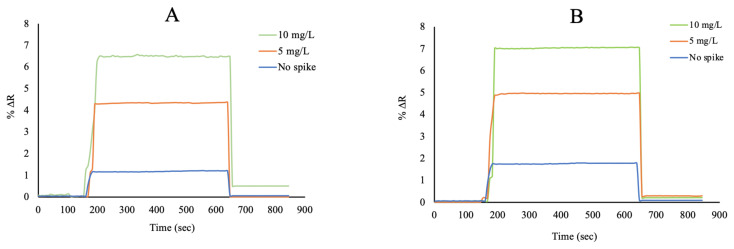
Kinetic analysis for curcumin detection in tea sample (A) and powder sample (B).

Real sample analyzes were carried out to demonstrate the accuracy of the curcumin imprinted SPR nanosensor. Kinetic analyzes for the detection of curcumin were performed by extracting curcumin from tea and powdered samples. After the extraction, kinetic analyzes were performed with the supernatant pellet portion. Curcumin solution (5 and 10 mg/L) was added to tea and powder extracts as indicated in Table 3. The obtained extract from tea and powdered was added from curcumin aqueous solution at concentrations of 5 and 10 mg/L. Firstly, the curcumin imprinted SPR nanosensor was first equilibrated with phosphate buffer (pH = 7.4) for 3 min. Then, tea and powdered extract spiked at concentrations of 5 and 10 mg/L were applied to the SPR system for 8 min. The removal of curcumin molecules from the curcumin imprinted SPR nanosensor surface was carried out with 0.5 M NaCI solution for 3 min (Figure 9). As a result, the curcumin amounts in real samples were calculated according to the equation y=0.4218x+1.0889 as shown in Table 3.

Liquid chromatography-tandem mass spectrometry (LC-MS) (Thermo Scientific TSQ Quantum Access Triple Quadrupole Cihaz, San Jose, CA, USA) was used for the determination of curcumin concentration in spiked samples. LC-MS separation was achieved by C18 reverse phase column (2.1×50 mm, 1.7 μm) Agilent Technologies, Santa Clara, CA). The mobile phase A was composed of 0.1% formic acid in water, and the organic mobile phase B was acetonitrile (ACN). All analyses were performed at 100 μL/min flow rate [39]. The spiked samples were injected into the device at 20μL. The total run time for LC-MS analysis was 18 min. Table 3 shows the recovery (%) for determining the reliability and accuracy of both the curcumin imprinted SPR nanosensor and LC-MS analysis results. The results show that the curcumin imprinted SPR nanosensor is quantitative, precise, stable, and sensitive for curcumin detection in tea and powder samples, which were based on the accuracy of SPR nanosensor and LC-MS results.

### 3.4. Investigation Repeatability of SPR Nanosensors

**Figure 10:**
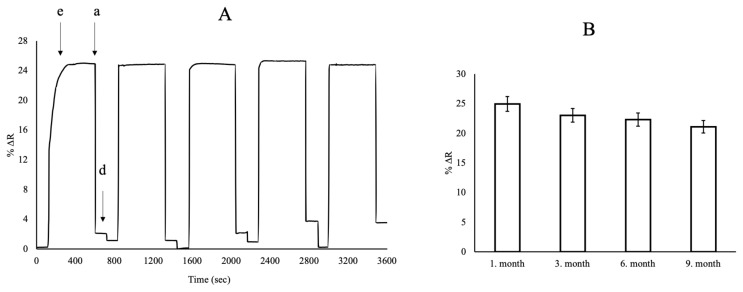
Repeatability (A) and short-term and long-term stability (B) of the curcumin imprinted SPR nanosensors (e: equilibration, a: adsorption, d:desorption cycles).

The repeatability of the curcumin imprinted SPR nanoensor was tested using the same curcumin concentration solution (50 mg/L) in five times equilibration, adsorption, and desorption cycles. The curcumin imprinted SPR nanosensor was equilibrated with the phosphate buffer (pH = 7.4) for 3 min. The curcumin solution with a concentration of 50 mg/L was applied to the SPR system for 8 min. The removal of curcumin from the curcumin imprinted SPR nanosensor surface was carried out with 0.5 M NaCI solution for 3 min in Figure 10A. After 9 months, the storage stability and efficiency of the curcumin imprinted SPR nanosensor was tested in the presence of 50 mg/L curcumin solution (Figure 10B). In addition, the repeatability of the curcumin imprinted SPR nanosensors at different times was analyzed with curcumin solution at a concentration of 50 mg/L during the first month, third month, sixth month, and ninth month (Figure 10B). These results indicated that the curcumin imprinted SPR nanosensor was not only reusable but also stable without degradation over time. According to repeatability results, the curcumin imprinted SPR nanosensor showed that it works with 84.57% efficiency at food analysis.

## 4. Conclusion

Nowadays, excessive use of curcumin, which has therapeutic and anti-inflammatory properties against many diseases with different mechanisms, this situation threatens human health negatively. At the same time, in terms of food safety imitation and adulteration of curcumin in products on the market can lead to negative consequences. Therefore, the detection of curcumin is so important for both human health and food safety. In this study, a sensitive, reliable and fast method has been developed for the determination of curcumin. As a result of the kinetic analysis performed with the curcumin imprinted nanosensors, LOD and LOQ were found to be 0.0012 mg/L and 0.0040 mg/L, respectively. According to selectivity studies, the resonance frequency change of the curcumin imprinted SPR nanosensor is higher than competing molecules as tartrazine and sunset yellow. Selectivity results showed that the cavities formed in the curcumin imprinted nanoparticles were sensitive to curcumin rather than tartrazine and sunset yellow. The repeatability of the curcumin imprinted SPR nanosensors were determined as 84.57%. In this study, an innovative, practical, and rapid analysis with SPR nanosensor for the detection of curcumin is presented. We aimed to develop an analysis method with good stability and sensitivity for the detection of curcumin. The results of this study showed that the curcumin imprinted SPR nanosensor was a useful method for food and drug application.

## Figures and Tables

**Table 1:** Comparison of curcumin detection methods.

Method	Detection range	Sample	LOD	RF
Fluorescent sensor	0.0576–2.3026 mg/L	Drinking water, ground ginger, curry powder, ginger juice, mustard, capsicum, Chinense juice	0.0067 mg/L	30
Fluorescent nanosensor	0.0115–4.0295 mg/L	Mustard, ginger, red paprika and turmeric powder	0.0044 mg/L	31
Fluorescent nanosensor	0–172.695 mg/L	Tap water and mineral water	0.0078 mg/L	32
Fluorescent sensor	0.1524–2.2865 mg/L	Curcumin solution	0.0469 mg/L	33
Fluorescent chemnanosensor	0.0576–2.3026 mg/L	Ginger juice, curry powder, mustard, ground ginger, radix curcuma	0.0093 mg/L	34
Fluorescent sensor	-	Ginger	As low as 0.1324×10^−^^6^ mg/L	35
Paper sensor	0–299.338 mg/L	Orange juice and curry solution	0.015 mg/L	36
Voltametric sensor	0.0230–36.8416 mg/L	In foods in the presence of vitamin B9	0.0092 mg/L	37
Voltametric sensor	0.0230–0.1151 mg/L	Natural food supplement	0.0031 mg/L	38
Surface plasmon resonance nanosensor	0.01–150 mg/L	Curcumin tea and curcumin powder	0.0012 mg/L	This study

**Table 2:** The selectivity and relative selectivity coefficients for CUR, TAR and SY molecules for the curcumin imprinted and the non-imprinted SPR nanosensors.

	MIP Nanosensor		NIP Nanosensor		
Molecules	ΔR	k	ΔR	k	k′
**CUR**	25.07	−	1.51	−	−
**TAR**	4.51	5.56	1.32	1.14	4.88
**SY**	1.09	23.00	1.25	1.21	19.01
**CUR+TAR**	20.59	1.22	1.44	0.69	1.77
**CUR+SY**	22.10	1.13	1.31	1.15	0.98
**CUR+TAR+SY**	23.15	1.08	1.49	1.01	1.07

**Table 3:** The recoveries of curcumin in tea and powder real samples at the curcumin imprinted SPR nanosensor.

Food samples	Added Curcumin (mg/L)	Found Curcumin (mg/L)		Recovery (%)	
		SPR	LC-MS	SPR	LC-MS
**Tea**	5	4.92 ± 0.0082	4.91 ± 0.009	98.4 ± 0.630	98.35 ± 0.191
	10	9.96 ± 0.009	9.91 ± 0.01	99.68 ± 0.095	99.15 ± 0.100
**Curcumin powder**	5	4.93 ± 0.006	4.89 ± 0.008	98.5 ± 0.115	97.8±0.163
	10	9.91 ± 0.008	9.88 ± 0.005	99.1 ± 0.082	98.85 ± 0.057
